# Success in the Profession of Dentistry

**Published:** 1882-11

**Authors:** F. W. Sage


### Success in the Profession of Dentistry.

F. W. SAGE, D. D. S.

We are now about to consider the question of success in practice, not especially as regards satisfaction given to our patrons, (an all-important requisite, of course,) but as regards the best means for building up a practice, securing the highest respect of the community for ourselves and for our profession, and winning the rewards to which, after a full and thorough performance of duty, we are justly entitled. The young dentist just entering upon his career, actuated by a lofty purpose and fired with enthusiasm, will need to exercise remarkable foresight, to avoid the errors and pitfalls into which many of his predecessors have fallen. If we were required to name a single quality which, superadded to the requisites of genuine ability, diligence, thoroughness, patience, and a pleasing address, is essential to success, we should say *decision of character*. The man who, in any business defines for himself a course of action, pursuing it without deviation in spite of the disapproval of some, adhering tenaciously to what he believes to be right, will in the end establish himself most firmly in the confidence of the public, and best advance his own interests. So it is our honest belief that the dentist who requires of all his patrons adequate compensation, having the nerve under all proper circumstances, to turn away many whose ideas of compensation do not accord with his own, will not only be more likely to reap a larger harvest but will actually extend his sphere of usefulness. He will need, it is true, to use careful discrimination, in order to avoid the imputation of selfishness and avarice, since many who highly appreciate skillful services are really unable to pay for them all they are worth. There is, however, nothing in the code of ethics of the profession which requires the dentist to establish fees subject to the approval of his individual patrons. The spirit of competition in fees seems to prevail to a greater extent in the dental profession than in either the medical or legal profession. People show but little disposition to "shop" when they go to consult a physician or a lawyer. Why is this so? Briefly stated, we believe the answer is to be discovered in the fact that there is a more distinct apprehension on the part of the public that a doctor's or a lawyer's *experience* is his capital and stock in trade, and therefore there is less disposition to question the right of these professionals to place their own estimate on the value of their services. If this answer be not enough to cover the ground we will elaborate it a little. Several things must be taken into consideration before we can fully understand why there should be a difference in the public's estimate of *our* privilege and that of the lawyer or doctor, in this respect. First of all, the public—the masses, at least—have hardly yet been brought to recognize dentistry as a profession. Those who accord to the members of the profession a more important function than that of mere "tooth pulling," are still slow to discover and still more slow to remember that the surgical and the conservative aspects of dentistry at this day indicate a degree of progress towards perfection such as justly entitles dental practitioners to all the dignity and honor of professional men. In the estimation of the masses a filling is a filling and the frequently repeated question, "What is your charge for gold fillings, doctor?" indicates only too often a disposition to make a "bargain" in advance, and to place limitations upon the dentist, on whose *skill* rather than on whose *integrity* confidence is reposed. That few have any appreciation of the time, labor, and pains-taking required of the operator, why need we argue? What need of inquiring further to ascertain whether distinctions as to qualifications to practice dental *surgery* find recognition in the minds of the masses?

Is not the idea which with many prevails, that one dentist is as good as another, sufficient to account for the "shopping" mania? We are compelled to accept the mortifying fact that the public do not sufficiently discriminate and give credit where it is due. Time will teach the people something. We mean no aspersion upon honorable, self-respecting men in the profession when we say that the responsibility for this state of affairs rests largely upon the dentists. There are plenty of men of fair attainments, who place so low an estimate on their attainments as *professional*, that they are contented with as much or as little respect as their patrons are disposed to accord them. They are ready to subordinate their own ideas of adequate compensation, to the caprice of every comer. Consequently, the opinion which the public holds of them is but the reflection of the opinion which they hold of themselves. Resolving one day to exact proper remuneration for his services in the future, uncertain rumors of the nearest rival's having lowered his prices, fill the mind of Dr. Timorous with gloom. His hours of solitude are occupied in studying up expedients to enable him to compete successfully with Dr. Flash, on his own plane, instead of being occupied in studying how he shall perfect his operations and so beat Dr. Flash by virtue of a genuine superiority. It seems superfluous to say that the number of Dr. Flashes in the profession who are really competent and conscientious, is exceedingly small, and that he who holds steadfastly his way and leaves the public to find out this fact will lose less than he who gets into a flutter and keeps an eye on his neighbor's barometer instead of on his own.

Decision of character helps a man wonderfully, under such petty trials as this. Let the young dentist form as nearly as may be possible, a definite idea of what he ought to be paid, and then *stick to it.* He will be sure to make some enemies. But *that* he will do whether he charges much or little. He who suffers himself to be easily influenced in this matter by what people say, will be as much embarrassed in trying to please all, as was the old man in the fable of Æsop, who started with his son to drive an ass to market. The story goes on to relate how, (to please the different groups of people they met on their journey,) the old man first rode the ass, then the son, and finally both rigged a pole to carry the ass.

But the young practitioner who means sturdily to stand by his fee-bill must be sure to do his work a little better than any one else does. If he chooses occasionally to work for less than the regular fee, he will act wisely, in almost every instance, in respectfully informing the recipient of the favor, that it is a gift. This he owes himself as a precaution against future imposition. The best of people are often thoughtless, and in the event of a failure, (for the best operators do fail, not unfrequently either,) the dentist will be expected, by four people out of five, to make good the failure, without additional charge. Possibly we are somewhat cynical, this evening, but be that as it may, the precaution we suggest will save trouble and is a good one. Don't be too much afraid of placing your patient in the attitude of a suppliant, young man. It won't cost him any more in the matter of humiliation than your lowering your fee costs you in actual cash. Another thing: never base any promises whatever on the presumption of your work's being everlastingly durable. If we did not take it for granted that you are bound by the ethics of the profession, we should say simply, *never warrant your work.* Nothing cheapens the dentist more, in the eye of the public, than the cringing, apologetic way in which some men go to work and re-insert a filling, *gratis*, say two years after date of first insertion. Say what we may, until natural conditions change, until teeth get ready to quit breaking away from fillings, until people quit having "sucker" mouths and masseter muscles like the tendons in a mule's hind leg, the dentist cannot always be certain that his work will last. *Promise nothing.* Let the people understand that you are careful and conscientious, and although you may see a few go away from your door because you will not "insure" your work, you will be the gainer in the end. But withal, if you find that through a heedless error or an inexcusable lack of judgment your work has failed, let your patient know that you are not inclined to shirk just responsibilities. We are not speaking from experience in all that we have said. We graduated in a class of first-class operators, each member of which had enjoyed the pupilage of the only really first-class operator in the country. Many of our class-mates brought to the college a fund of invaluable information, and it leaked out that one or two had been secretly appointed as "alternates" in case any member of the faculty should fail. The hints we have presented are gathered from the experience of some fellow-practitioners, who, unlike ourself, do not enjoy the exclusive patronage of high toned, long-pursed patients.

Again; do not assume a look as if you had just been detected in the act of leaving a gambling den by way of the back alley, when a patient returns to you with a filled tooth aching. Smile pleasantly, and say, "That's *too bad.*" It is just as well, (if you happen to be operating at the chair when he comes in,) to keep him waiting in the other room for twenty minutes, while you fool around finishing a filling a good deal more than is necessary so that your patient will think he is getting his money's worth. You may occasionally inquire, over your shoulder, if it "hurts now," humming a tune cheerfully all the while. This will show the new-comer about how much idea you have of assuming responsibility for the tooth's aching, and will make him feel what a privilege it is to be taken into your sympathy at a time when you are so very busy. At the same time, it will convince the patient in the chair that the fellow waiting must have been guilty of some gross indiscretion, to be suffering with the tooth-ache so soon after having enjoyed the privilege of your services. The patient sufferer will probably give an exaggerated wail of anguish every once in awhile, but you mustn't let that disconcert you. Ask him if his sister enjoyed her trip to the sea-shore. You might go out and borrow his knife to whittle a wedge, just by way of showing any others who may be waiting, what perfect good feeling exists between you. After your patient has vacated the chair, and you have called your boy to empty the spittoon, and have washed your hands, and gone down stairs to see if the semi-weekly paper has come, you finally seat the sufferer in the chair and look at the tooth. Then you heave a sigh of relief and say, "Is *that* all?" and at once proceed to paint the gum with iodine, whistling composedly, meanwhile. It is well to inquire as to the state of his bowels. Finally dismiss him with an injunction not to stay out so late of nights, and to be careful about his diet for a few weeks. You may follow him to the door and advise him to call later, (after business hours,) to report progress. He may be depended upon to call as requested, when you at once proceed systematically to undermine the constitution of that nerve.

On general principles it is just as well to keep a patient waiting awhile, if you happen to be out of the office when he calls. We have known a dentist to throw away half of a fine cigar and hurry to wash out his mouth and beard the moment a patient was announced. Any thoughtful person will at once perceive that this is the height of indiscretion. Don't rush into the waiting-room as if you had not had a call for a week. If the caller wants a sitting immediately, you must look perplexed and say that you will send your boy to see if Miss M. will consent to postpone her appointment for an hour or two. Don't give him a second sitting for three days, at least, no matter if you haven't anything to do but pitch quoits in the back yard.

Now again, seriously: Do not allow your patients to cultivate the idea that payment for services once rendered confers on them the privilege of running in and annoying you every time a bit of meat lodges between two teeth. If a filling has not been properly contoured, attend to that. Some people act as if they thought a sight of their countenances was calculated to thrill your bosom with pleasure, recalling the time when they paid you a considerable bill. The best way to discourage these infringers upon your valuable time is to send in another bill, if a reasonable pretext offers. In the earlier days of the writer's—that is to say, rather, of the young dentist's practice, it is no uncommon thing for half a day to be taken up with profitless tinkering for people who never think of asking what is the fee and who are offended if a bill be sent in. The young man learns after a few years, that there is such a thing as being too good-natured. It is not difficult to understand why people expect the dentist to make good all losses, while they submit with less complaining to additional charges made by the tailor, the dressmaker, and the mechanic. It is, in the first place, for the reason that they probably feel that the dentist ought to pay the penalty for their repeated submission to a trying ordeal. It is, in the second place, the fruit of the teaching of over-confident men in the profession, who, in order to secure patronage, promise more than circumstances warrant, or who, from a mistaken notion of what they owe the unfortunate patient, (by way of cheering him and allaying misgivings as to future trouble,) assure him of immunity. It requires some moral courage to tell the shrinking patient, frankly, that the pain and inconvenience to which he must submit, and the expense which will be involved, may not after all, accomplish the result desired of saving him from future suffering. Such candor would lose to the operator many a fee. Just here we labor under a disadvantage. The patient has the alternative before him, of gas and an artificial denture. The surgeon's patient has usually no similar alternative.

But the principle upon which the public presume to hold the dentist responsible, is wrong, and in justice to the profession should be righted. Each one holds in his own hands the means of preventing or of correcting this unjust assumption.

Decision of character is above all indispensable to enable the dentist to control his patients. If Master Charlie fails to keep his second appointment, prompt him by every means in your power, regardless of every consideration which stands in the way of your doing your whole duty to him. If Miss Nellie hesitates to take the time necessary for such preliminary treatment as may be required, it is then your privilege and duty to hesitate to undertake the case. Be assured, you will lose nothing by adhering to your principles.

Compel respect and you will conquer success.

